# Supplementary material for: Cloud BioLinux: pre-configured and on-demand bioinformatics computing for the genomics community
Source: BMC Bioinformatics. 2012 Mar 19;13:42. doi: 10.1186/1471-2105-13-42 (PMC3372431; doi:10.1186/1471-2105-13-42)
Supplement: Additional file 1 — Supplementary 1 Cloud BioLinux software documentation in the form of a mini, self-contained website. Users need to download and uncompress the .zip file, and open through a web browser the "index.html" file available on the main directory. (ZIP 1823 kb). [file 1471-2105-13-42-S1.ZIP › Cloud-BioLinux-Package-Documentation/docs/clcworkbench.html]

Bio-Linux Software Documentation Pages

Back to search form

## clcworkbench

|  |  |
| --- | --- |
| Name | clcworkbench |
| Description | **CLC Workbench** is a graphical environment in which you can carry out many basic bioinformatics tasks. Some of the functionality available is listed below:   - Multiple alignment of DNA, RNA, and proteins- Consensus sequence determination- Conservation score along alignments- Basic gene finding- Translation from DNA to proteins (all genetic translation tables)- Reports with residue composition, molecular weight and iso electric point (for proteins)- Neighbor-joining and UPGMA phylogenies- Restriction site analysis and viewing- Create reverse complement- Shuffle sequence |
| Homepage | http://clcbio.com/index.php?area=software&section=free |
| Remote Documentation | http://clcbio.com/files/free\_finalManual\_A4.pdf |
